# Capturing Correlation Effects in Positron Binding to Atoms and Molecules

**DOI:** 10.1021/acs.jctc.4c00727

**Published:** 2024-09-17

**Authors:** Shiv Upadhyay, Anouar Benali, Kenneth D. Jordan

**Affiliations:** † Department of Chemistry, University of Washington, Seattle, Washington 98195, United States; ‡ Department of Chemistry, University of Pittsburgh, Pittsburgh, Pennsylvania 15218, United States; § Computational Sciences Division, Argonne National Laboratory, Lemont, Illinois 60439, United States

## Abstract

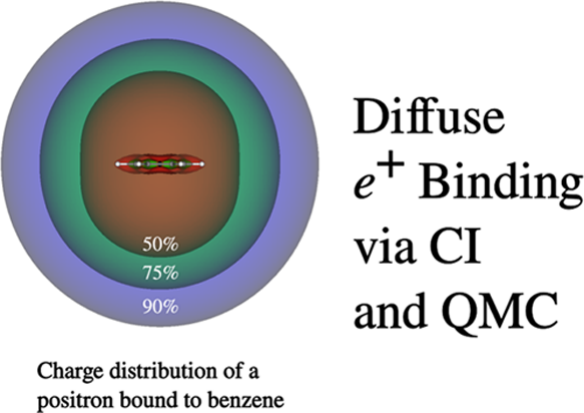

A major challenge in contemporary electronic structure theory involves the development of methods to describe in a balanced manner the contribution of correlation effects to energy differences. This challenge can be even greater for multicomponent systems containing more than one type of quantum particle. In the present work, we describe a flexible code for carrying out self-consistent field and configuration interaction (CI) calculations on multicomponent systems and use it to generate trial wave functions for use in diffusion Monte Carlo (DMC) calculations of the positron affinity of Be, Be_2_, Be_4_, Mg, CS_2_, and benzene. The resulting positron affinities (PAs) are in good agreement with the best values from the literature.

## 1 Introduction

The origins of multicomponent methods can be traced to Thomas for the description of quantum nuclei.^1−4^ Two other main application areas of multicomponent methods are treating electrons and nuclei (generally protons) on equal footing,^5−109^ and in describing the binding of positrons to atoms and molecules.^110−134^

In this work we describe the implementation of a flexible computer code for characterizing multicomponent quantum systems and apply it to generate trial wave functions for subsequent diffusion Monte Carlo (DMC) calculations of the positron affinities (PAs) of several test systems. The binding of a positron to an atom or molecule occurs via a diffuse nonvalence orbital. Electrostatic interactions alone suffice to bind a positron to anions and to sufficiently polar molecules. Here we are interested in more challenging systems in which electrostatic interactions alone do not suffice to bind the positron and for which correlation between the positron and the electrons is fundamental to the binding. In this sense the situation is similar to that for nonvalence correlation-bound (NVCB) anions.^135−138^

The binding of positrons to atoms, molecules, and clusters can be described by multicomponent configuration interaction (CI) methods, which are a generalization of traditional quantum chemistry CI methods. Due to the basis set demands, it is computationally challenging to obtain well converged values of PAs for large polyatomic systems when using such methods. We note that over the years, there have been great efforts to push multicomponent CI methods to generate accurate, but computationally tractable approximations to circumvent this issue.^100,102,120,139,140^ An alternative approach for circumventing the basis set issue in calculations on positron-atom and positron-molecule complexes is to employ the DMC method, which should be ideally suited for characterizing these complexes, provided that suitable trial wave functions for describing the nodal surface for electron exchange are employed.

In this work we use the DMC method to calculate PAs of Be, Be_2_, Be_4_, Mg, CS_2_, and benzene. For these species the positron does not bind with the simplest wave function, a product of a positron orbital and a single Slater determinant for the electrons. In fact, with such a wave function and the use of a flexible basis set, the positron occupies a discretized continuum (DC) orbital. The collapse of the positron orbital onto a DC level could be problematical for DMC calculations of PAs as such a trial wave function would not capture the impact of the positron on the electron–electron nodal surface. This issue is addressed in the present study by examining the suitability of various CI trial wave functions for describing positron binding in DMC calculations. We also report a CI result for HCN which has been the subject of several prior theoretical studies.^141−143^

While experimental values of the PAs of CS_2_ and benzene exist, experimental results do not exist for Be, Be_2_, Be_4_, and Mg. Nonetheless, we consider calculations of the PAs of these species to be particularly valuable due to the large variation in their polarizabilities.

## 2 Theory

The computer code (named Polyquant available at www.github.com/shivupa/polyquant) that we developed for characterizing systems with two or more types of quantum particles is described in this section. When starting with a wave function that is a product of Slater determinants for each type of quantum particle of interest one can derive separate Fock operators for each type of quantum particle. The elements of the Fock operators in an atomic orbital basis are given by
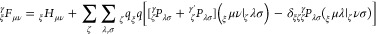
1In this expression, γ ∈ {α, β}, where α and β denote up and down spin respectively; γ′ is the opposite spin from γ; ξ and ζ run over the quantum particle types; and μ, ν, λ, σ are atomic orbital indices. *F* is the Fock operator, and *H* is the one electron Hamiltonian. *P* refers to the one-particle density matrix in an AO basis. In eq 1,  denotes the two-particle integral in chemist’s notation over the Coulomb operator. We note that both atomic orbitals in the bra or ket are labeled with a single particle label since mixed particle exchange is not possible.

For a given particle type one can employ either a spin-restricted or spin-unrestricted treatment. In the spin-restricted case only a single Fock operator is constructed for the particle type of interest, but the normalization of the density matrix is kept the same as for the unrestricted case. To expound on this notation, the restricted Fock operator for the electrons in a multicomponent electron-positron calculation is given as
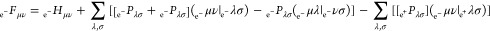
Separate atomic orbital bases sets are used to describe the electrons and the positron. It should be noted that although the basis sets are referred to as atomic orbital, the functions can be centered at off-atom sites.

Each quantum particle contributes
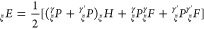
2to the total energy, which is given as the sum of the individual contributions from each particle type plus the nuclear repulsion energy, *E*_nuc_
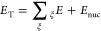
3

### 2.1 Convergence of the Self-Consistent Field Equations

A standard multicomponent approach converges the density in a stepwise fashion, e.g., for an electron-positron calculation, converging the electron density followed by converging the positron density and repeating this cycle until overall self-consistency is achieved.^144^ It has been reported that iterating the densities simultaneously until self-consistency is achieved is more efficient.^144^ In this work, we iterate the densities simultaneously, but with an important distinction, we do not turn on interactions between quantum particle types until the noninteracting densities are converged. For the species for which the positron is not bound in the Hartree–Fock (HF) approximation this led to improved convergence.

### 2.2 Self-Consistent Field Implementation Details

The self-consistent field (SCF) equations are solved using a direct algorithm. Incremental Fock formation has been implemented, as well as direct inversion of the iterative subspace (DIIS) extrapolation to accelerate convergence. Both symmetric and canonical orthogonalization are implemented, with a preference for canonical orthogonalization as it allows for reduction of the size of the basis set by removing the linear dependencies in the atomic basis. Abelian point group symmetry can be exploited through an interface with the library libmsym.^145^ Furthermore, spherically symmetric systems can utilize full SO(3) symmetry during the SCF procedure.

### 2.3 Configuration Interaction

The multicomponent configuration interaction ansatz is formed from a linear combination of products of Slater determinants (SDs)
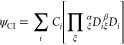
4In practice, this expansion is truncated at a certain excitation level relative to a mean field solution. For example, a multicomponent CI expansion consisting of all single excitations relative to a reference determinant is given as
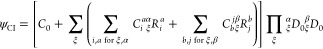
5where the excitation operators  and  perform an excitation for quantum particle type ξ and spin α(β) from hole *i*(*j*) to particle *a*(*b*). To make things explicit, we define the excitations in the following way

1. A maximum overall excitation level is specified.
2. For each particle type, a maximum excitation level is adopted for each spin along with a combined maximum excitation level.

This allows for highly flexible configuration interaction calculations. For example, for the positron complexes this allows for a restricted single double configuration interaction (rSDCI) with at most excitation of one electron.

#### 2.3.1 Diagonalization to Solve the CI Eigenvalue Problem

The CI eigenvalue problem
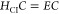
6is solved using the Davidson diagonalization method.^146^ For small CI spaces, the Hamiltonian matrix can be explicitly constructed, but for larger spaces a determinant-driven direct CI method in which the Hamiltonian matrix is never explicitly constructed is employed. This enables large CI calculations for systems where the CI matrix would not fit in memory. Central to this method is the formation of the matrix-vector product in which the Hamiltonian is contracted with a trial vector
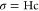
7In standard electronic structure theory, the formation of the σ vector is a well-studied problem and several efficient algorithms have been developed.^147−149^

Since the CI problem of interest here is generalized by excitation level for each particle and spin type, the standard methods developed to treat the complete set of excitations in a particle and spin space (or the complete set of excitations within an active space) are not applicable. Below we describe the less efficient, but more flexible, method used here. The sigma vector for one quantum particle type, *A*, involves 3 terms
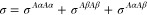
8and similarly for two quantum particle types, *A* and *B*
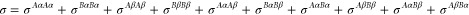
9The general number of terms, *N*_σ_, in a σ vector formation for a *N* quantum particle type system is
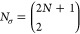
10

The single quantum particle sigma formations require different algorithms for the same spin (σ^*A*α*A*α^ and σ^*A*β*A*β^) and opposite spin (σ^*A*α*A*β^) cases. Similarly for the two quantum particle sigma formation, there are two classes of contributions:

1. same particle and same spin contributions (σ^*A*α*A*α^, σ^*A*β*A*β^, σ^*B*α*B*α^, σ^*B*β*B*β^)
2. different particle or different spin contributions (σ^*A*α*A*β^, σ^*B*α *B*β^, σ^*A*α*B*α^, σ^*A*β*B*β^, σ^*A*α*B*β^, σ^*A*β*B*α^)

One may form the overall sigma vector with minimal modification to standard electronic structure programs by forming the contributions from each particle type independently and then forming the mixed particle terms as was done by other groups in the past or by implementing generic kernels for each of the above classes as is done in this work. The latter option allows for future generalization to multiple interacting particle types since for three or more interacting particle types the different sigma contributions will be at most two body and thus fall into one of the two categories above. In the current iteration of the code, the matrix vector product is formed in a single step that scales as , where *N*_det_ is the total number of determinants and the other factors of *N* are the numbers of unique spin determinants for each pair of particle and spin types. Additionally, natural spin orbitals can be constructed from the one-particle reduced density matrices from the CI expansions in a state specific way. These natural orbitals can be used in constructing more compact trial wave functions for subsequent QMC calculations.

CI calculations in a determinantal basis are not guaranteed to produce states that are spin eigenfunctions unlike CI calculations in a configuration state function basis. The standard solution to this is to implement a spin purification scheme in either a first or second order form.^150,151^ Both approaches have been implemented in Polyquant to enforce proper spin states for problematic cases.

#### 2.3.2 Configuration Interaction Implementation Details

The storage and processing of determinants is central to the configuration interaction code. We represent determinants using unsigned 64 bit integers for each particle and spin type. The integers are zero padded from the left until the entire 64 bit integer is filled. These integers are stored in a list to represent a single spin determinant, . A list of unique determinants is stored for each particle type and spin type. The complete variational space is then stored as an unsorted map between a tuple of indices into the unique spin determinant lists and the index of the determinant made of those spin determinants in the variational space.

To speed up the Hamiltonian construction or sigma vector formation, we store a list of unique single and double excitations for each unique particle and spin type. For example, for a spin determinant , we store all connected singles *j* in a two-dimensional (2D) list in row *i* and all connected double excitations *j* in another two-dimensional list in row *i*. The storage of double excitations is normally not done as the storage requirement grows quickly. However, for the same number of total determinants, the unique particle and spin determinant lists are shorter in a multicomponent calculation than an electron only calculation by nature of the product ansatz so we are able to accept the storage cost for the efficiency gain.

#### 2.3.3 Details of the Quantum Monte Carlo Calculations

In order to obtain DMC values for the positron affinities, separate DMC calculations were carried out on the species of interest in the absence of and in the presence of the positron. The trial wave functions used for these calculations involved a product of a Jastrow factor^152−154^ and a single configuration or multiconfiguration term generated using the Polyquant code described above. Hereafter we will use the designations single determinant (SD) and multideterminant (MD) for both the calculations with and without the positron, although the latter are comprised of products of Slater determinants for the electrons and an orbital for the positron. The electron Jastrow factors have both one-and two-body terms. In the calculations involving a positron, there is also an electron-positron two-body contribution. The Jastrow factors explicitly include the electron–electron and electron-positron cusps. A B-spline form of the one and two body Jastrow factors was used with the radial cutoff fixed at 10 Å. The electron–nucleus and positron-nucleus cusps conditions were enforced by cusp-corrected orbitals. The parameters in the Jastrow factors were optimized using the variational Monte Carlo (VMC) method using energy minimization. The majority of the DMC calculations on the systems without the positron were carried out for time steps of 5 × 10^–4^, 1 × 10^–3^, and 2 × 10^–3^ a.u., with linear fits being used to extrapolate energies to zero time step and utilized 10,000 walkers with 800, 400, 200 blocks of 1000 steps for the 5 × 10^–4^, 1 × 10^–3^, 2 × 10^–3^ a.u. time steps, respectively. The DMC calculations with a positron present required much more sampling due to the diffuse charge distribution of the positron, and, with the exception of CS_2_, utilized 10,000 walkers with 200, 100, 50 blocks of 20000 steps for the 5 × 10^–4^, 1 × 10^–3^, 2 × 10^–3^ a.u. time steps, respectively. For CS_2_, the DMC calculation with a positron utilized 24,000 walkers with 100 blocks of 25,000 steps for a single (1 × 10^–3^ a.u.) time step. The VMC and DMC calculations were carried out using the QMCPACK code.^153,154^ Although the focus is on the results of DMC calculations, we also report the PAs obtained from the trial wave functions themselves. It should be noted that all CI calculations reported in this study made use of the frozen-core approximation in which excitations from the occupied nonvalence orbitals were excluded.

## 3 Results and Discussion

Subsection 3.1 compares our result for the PA of HCN with other values from the literature. The PAs of Be, Be_2_, Be_4_, and Mg are considered in Subsections3.2–3.4. Subsections 3.5 and 3.6 summarize, respectively, our results and those of other researchers for the PAs of benzene and CS_2_.

### 3.1 HCN

We discuss the binding of a positron to HCN before turning to the more difficult case of nonvalence correlation-bound positron containing systems. Because the dipole moment of HCN at its equilibrium geometry is 3.01 D,^155^ the positron will bind in a single configurational treatment if a sufficiently flexible basis set is employed.^142^ I.e., electrostatics alone suffice to bind the positron to HCN, although inclusion of correlation effects is important to obtain a quantitatively accurate value of the PA. For our calculation on HCN we employed the aug-cc-pVDZ basis set for the electrons and for the positron a basis set with 5s4p1d on C and N, 1s on H, and a 16s16p6d1f basis set on the rotational axis, 1 Å “behind” the N atom. For HCN at its experimental equilibrium geometry we obtain a PA of 0.4 meV in the HF approximation. This value is smaller than the recent large basis set result of Hofierka et al.^142^ who obtained a HF value of the PA of 1.95 meV. Our rSDCI calculations give for HCN a PA of 60 meV, significantly larger than the rSDCI value of 39.55 meV reported by Kita and Tachikawa.^143^ This reflects our use of a more flexible positron basis set. Many-body calculations using a large basis set with 18 off-atom sites for the positron basis functions gave a value of 73 meV for the PA.^142^ In addition, a value of 30 ± 5 meV has been obtained from DMC calculations using single configuration trial wave functions. At present it is not known whether the underestimation of the PA by the SD-DMC calculations is a limitation of the basis set used to represent the trial wave function or whether it reflects the need to employ a trial wave function that includes correlation effects. This is an issue that we plan to address in a future study.

**Figure 1:**
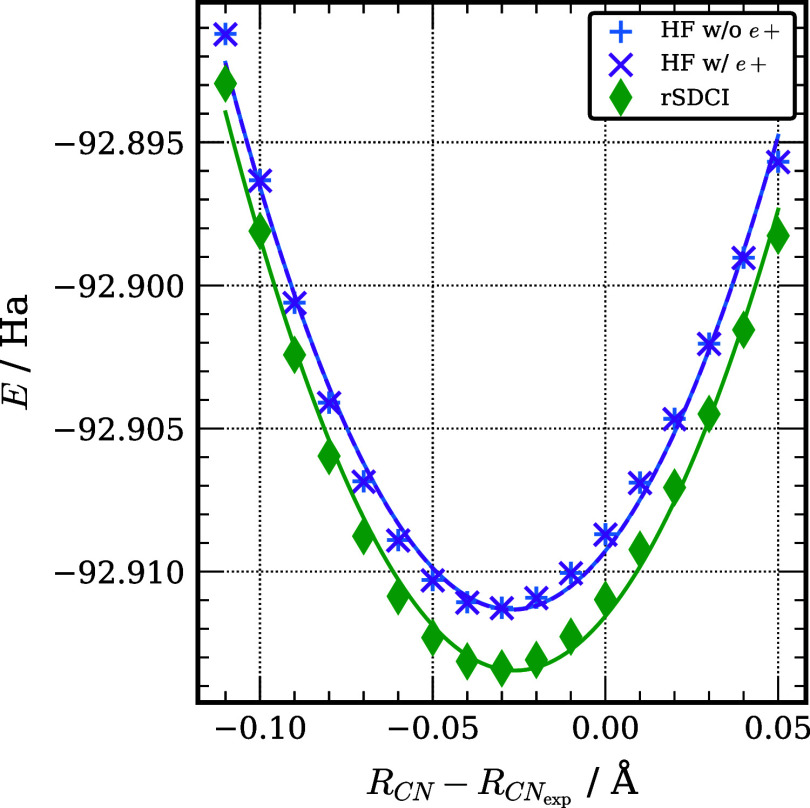
One dimensional scan of the CN potential energy surface of HCN. The HC bond distance is held constant at the experimental bond length of 1.064 Å.

Although the geometric dependence of the positron affinity is not the main focus of this work, we note that the rSDCI approximation can be useful for exploring this dependence. Figure 1 presents the one-dimensional potential energy curve for the CN stretch in [HCN; e^+^] with the HC bond being held constant at the experimental equilibrium value.

### 3.2 Be

We first consider positron binding to a Be atom as treated with Slater-determinant based methods. Our calculations on [Be;e^+^] employed the aug-cc-pVQZ^159^ Gaussian-type orbital (GTO) basis set for the electrons and an 11s8p6d6f3g GTO basis set for the positron. The positron basis set, which is given in the Supporting Information, was optimized in a series of rSDCI calculations on the positron complex. As expected, the HF calculations fail to bind the positron. On the other hand, the rSDCI approach, which employs the HF wave function for the isolated atom and a wave function that includes all single excitations plus those double excitations that involve simultaneous excitation of an electron and of the positron for the complex, gives a PA of 152 meV, appreciably larger than CI, stochastic variational method (SVM),^158^ and frozen-core stochastic variational method (FSVM)^158^ values reported in the literature and included in Table 1. Due to the use of the frozen-core approximation, our configuration interaction singles and doubles (CISD) calculations on Be is equivalent to a full configuration interaction (FCI) calculation. The corresponding calculation for [Be;e^+^] includes only triple excitations which involve the positron, which we call restricted single double triple configuration interaction (rSDTCI). These FCI calculations give a PA of 10 meV, appreciably smaller than previous CI, SVM, and FSVM values (see Table 1). The underestimation of the PA by our FCI calculations is not surprising given the limitations of the basis set used. Specifically, it is known that one needs to include much higher angular momentum functions in the basis set than employed here to accurately describe short-range electron-positron correlation and to achieve well converged results.^156^ Bromley and Mitroy obtained a value of 69 meV for the PA of Be from CI calculations using electron and positron basis sets with angular momentum functions up to *l* = 10.^156^ In addition, extrapolation of their results to the infinite basis set limit gave a value of the PA of 84 meV.^156^ The need to employ such large basis sets to obtain accurate PAs when using traditional quantum chemistry methods is precisely the reason that real space methods such as DMC are so useful in characterizing positron binding.

**Table 1:** PAs (meV) of Be, Be_2_, Be_4_, and Mg from Present Study Using Slater Determinant-based Methods

method^a^	Be	Be_2_	Be_4_	Mg
HF//HF^b^	–1.9	–56.5	–57.0	–1.9
HF//MD/rSDCI^b^	152.3	482.9	771.1	386.0
MD/CISD//MD/rSDTCI^b^	10.5	97.2		167.8
FCI	69.0^c^			
extrapolated FCI	84.0^c^			463.7^d^
FSVM	86.0^e^			460.7^d^
SVM	86.5^e^			

a The wave functions used in the absence of and in the presence of the positron are indicated to the left and right of the double slash, respectively.

b Present study.

c From Bromley and Mitroy.^156^

d From Bromley and Mitroy.^157^

e From Mitroy.^158^

**Figure 2:**
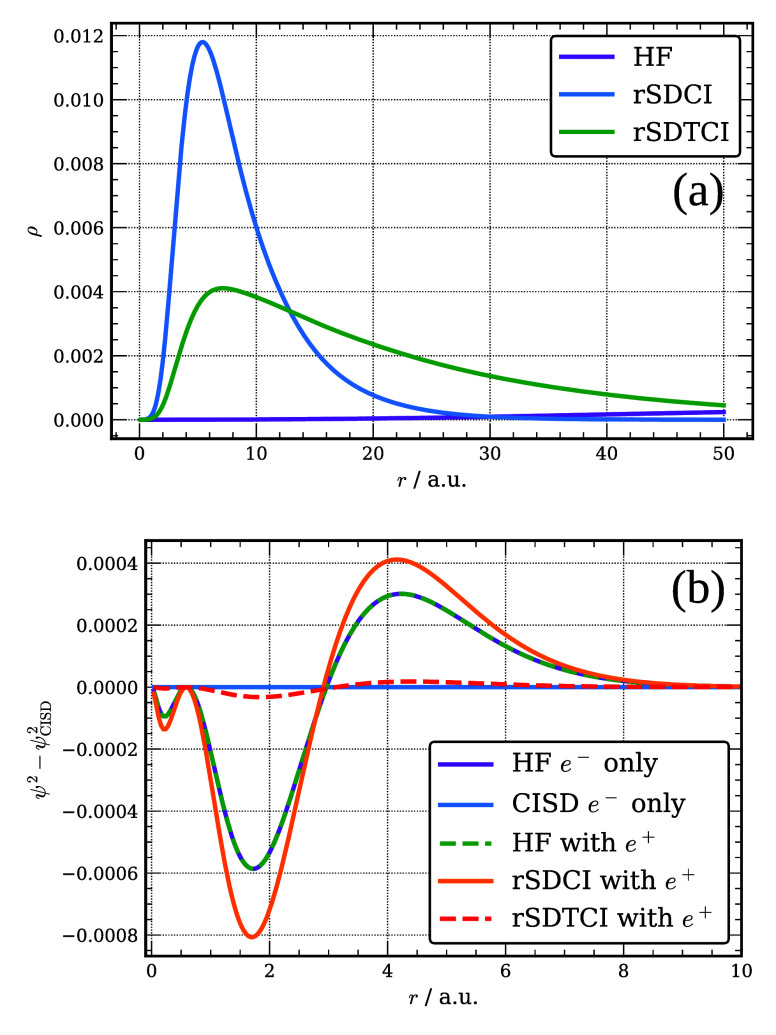
Radial distribution function of the Be 1s positron orbital (a) and differences in the distribution of the 2s electron orbital of Be and [Be; e^+^] described at various levels of theory relative to the FCI results for Be (b).

In Figure 2, we report the radial distribution of the positron 1s natural orbital (NO) of [Be;e^+^] as described by various Slater-determinant-based wave functions considered in this work. The figure also reports how the charge distribution of the electronic 2s NO from the various calculations on [Be;e^+^] differs from that from the FCI calculation on Be. As seen from this figure, the positron 1s orbital from the HF calculations is extremely diffuse as it has collapsed onto a DC level. The positron 1s NOs from both the rSDCI and FCI calculations on [Be;e^+^] are much more localized, with the associated radial distributions peaking at about 7.5 Bohr. The radial distribution of the positron 1s NO from the FCI calculations tails off much slowly with increasing distance from the nucleus than that from the rSDCI calculations, consistent with the weaker positron binding in the FCI calculations.

From part (b) of the figure it is seen that the 2s NO from the FCI calculation on Be is radially contracted compared to the 2s HF orbital. The presence of the positron as described by the FCI calculations on [Be;e^+^] causes a small radial expansion of the 2s electron NO. In contrast, the 2s electron NO as described by the rSDCI calculations on [Be;e^+^] is more radially extended than the 2s HF orbital of Be. We consider below the implications of these results for the DMC calculations of the PAs of Be.

**Figure 3:**
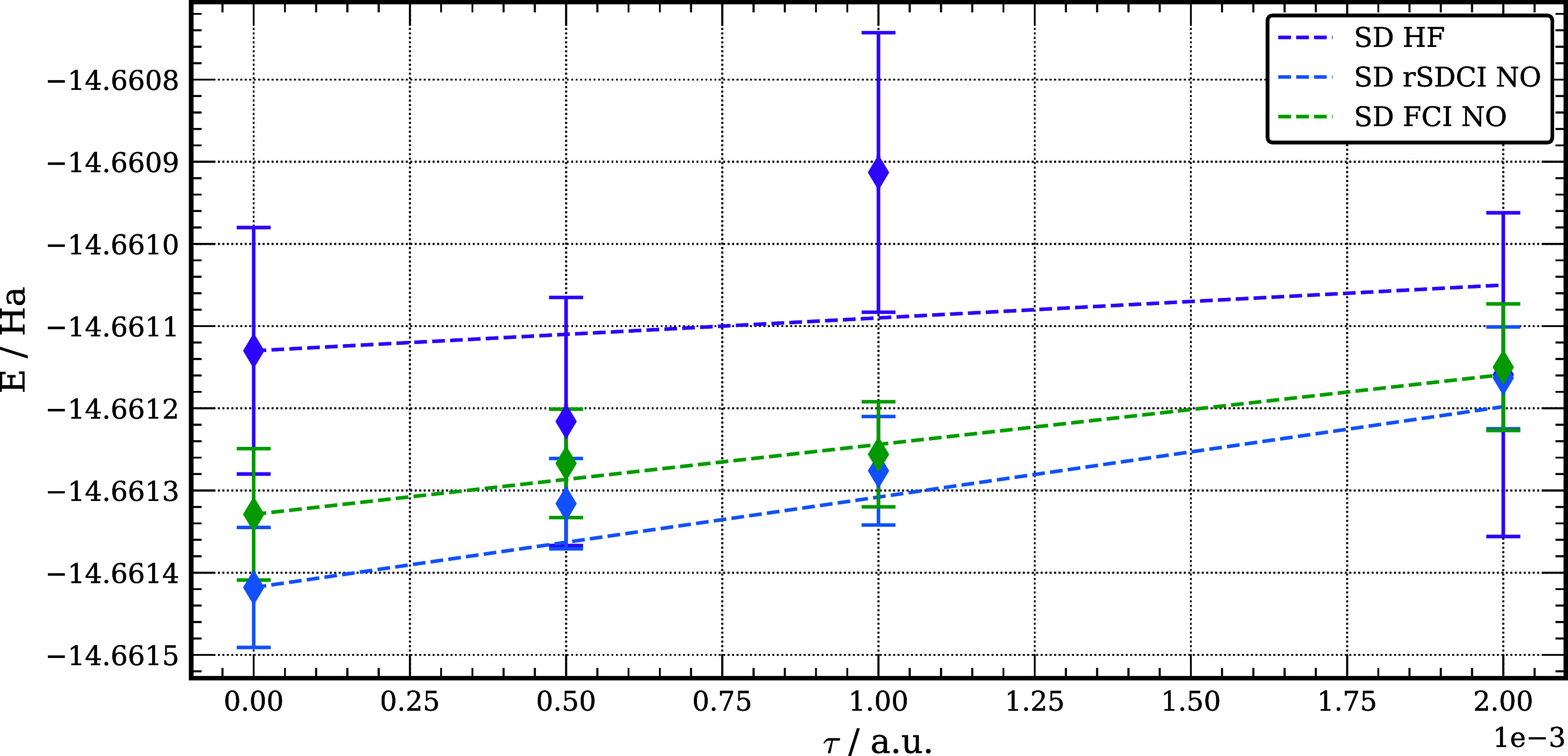
DMC energy versus time step for [Be;e^+^] using a SD trial wave function employing either HF orbitals, frozen core rSDCI NOs, or frozen core FCI NOs. Plot generated using Matplotlib.^161^

In our DMC calculations of the PA of Be three sets of SD wave functions were considered for the positron complex: one using HF orbitals, one using NOs from the rSDCI calculation, and one using the NOs of the FCI calculation. In the first two cases the trial function for the DMC calculations of the Be atom was a SD of HF orbitals and in the third case it was a SD of NOs from the FCI calculation on Be. The PAs obtained from the three sets of SD-DMC range from 95 to 104 meV and are statistically indistinguishable from one another and from the SD-DMC result of Mella et al.^160^ This result is expected since the nodal surface of Be as described by a single Slater determinant is independent of the orbitals used. As seen from Figure 3, the convergence of the DMC energy with time step is significantly better when using trial functions with a localized positron orbital than when using a positron orbital that has collapsed onto a DC level.

Our DMC calculations using FCI trial wave functions for both Be and [Be;e^+^] give a PA of 91 meV, which is quite close to the value obtained from the DMC calculations using HF trial wave functions. This result is within statistical error with the earlier SVM result, and is appreciable larger than the MD-DMC results of Mella et al.^160^ and Charry Martinez et al.^131^ The 33 meV PA result of Mella et al.^160^ (denoted 4D-DMC in Table 2) and the 34 and 54 meV PA values of Charry Martinez et al.^131^ used trial wave functions that accounted for the 2s^2^ → 2p^2^ configuration mixing which is known to be important in describing the nodal surface for the Be atom. Our DMC calculations using a HF trial function for the atom and the MD-rSDCI trial wave function for the positron complex, give a PA of Be of 151 meV. Thus, our calculations demonstrate that while the use of HF trial wave functions for Be and [Be;e^+^] results in a DMC value of the PA close to the best estimate, CI trial functions that are restricted in terms of classes of excitations that they include, e.g., those allowing only for the 2s^2^ → 2p^2^ electron excitations or a rSDCI treatment of the positron complex do not give balanced treatment of the electron nodal surfaces for Be and [Be;e^+^]. Trial functions including configurations in which both 2s electrons are excited are essential to describe the impact of the positron on the electron–electron nodal surface of Be.

**Table 2:** Comparison of PAs (meV) of Be and Mg from DMC Calculations

method^a^	Be	Mg
DMC: SD/HF//SD/HF^b^	96 ± 5	90 ± 27
DMC: SD/HF//SD/NO rSDCI^b^	104 ± 4	471 ± 14
DMC: SD/CISD NO//SD/rSDTCI NO^b^	95 ± 4	344 ± 23
DMC: SD/HF//MD/rSDCI^b^	151 ± 4	449 ± 32
DMC: MD/CISD//MD/rSDTCI^b^	91 ± 4	367 ± 35
SD-DMC^160^^c^	100 ± 5	457 ± 38
4D-DMC^160^^d^	33 ± 11	
DMC/PMO^e^	34 ± 8	
DMC/EPO^f^	54 ± 10	

a The wave functions used in the absence of and in the presence of the positron are indicated to the left and right of the double slash, respectively.

b Present work.

c From Mella et al.,^160^ used single determinant trial wave function.

d From Mella et al.,^160^ used four-determinant trial wave functions that allow for the 2s^2^ → 2p^2^ electronic excitations.

e From Martinez et al.,^131^ the trial wave function for the positron complex uses positron orbitals that do not depend explicitly on the electron orbitals.

f From Martinez et al.,^131^ the trial wave function for the positron complex uses positron orbitals that depend explicitly on the electron orbitals.

**Figure 4:**
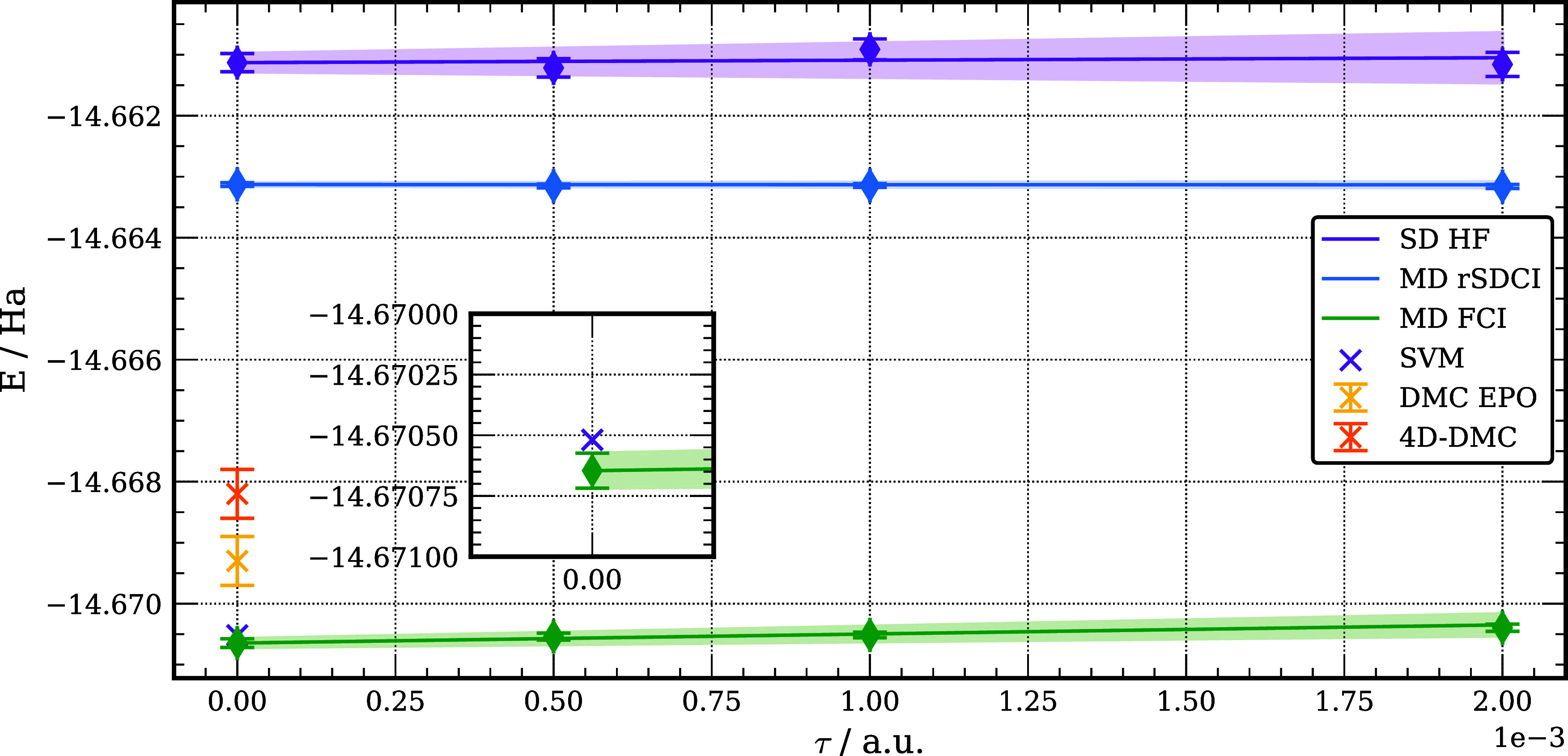
DMC energy versus time step for [Be;e^+^] with HF SD, rSDCI MD, and FCI MD trial wave functions indicate total energies of [Be;e^+^] from prior studies using the SVM,^158^ and DMC methods are also indicated.^131,160^ The shading represents the statistical uncertainty for methods involving extrapolation to zero time step.

To elucidate the importance of the nodal surface, we report in Figure 4 the DMC energies of the [Be;e^+^] system obtained in this study and in prior studies using different trial wave functions. Going from the HF to the rSDCI multideterminant trial wave function lowers the DMC energy of the complex by about 50 meV, but a much larger energy decrease (about 200 meV) results when using the FCI trial wave function. Significantly, it is seen from the figure that our DMC energy of [Be;e^+^] obtained using a FCI trial function is lower than that obtained in other recent studies using the DMC method and is essentially identical to the SVM result.

**Figure 5:**
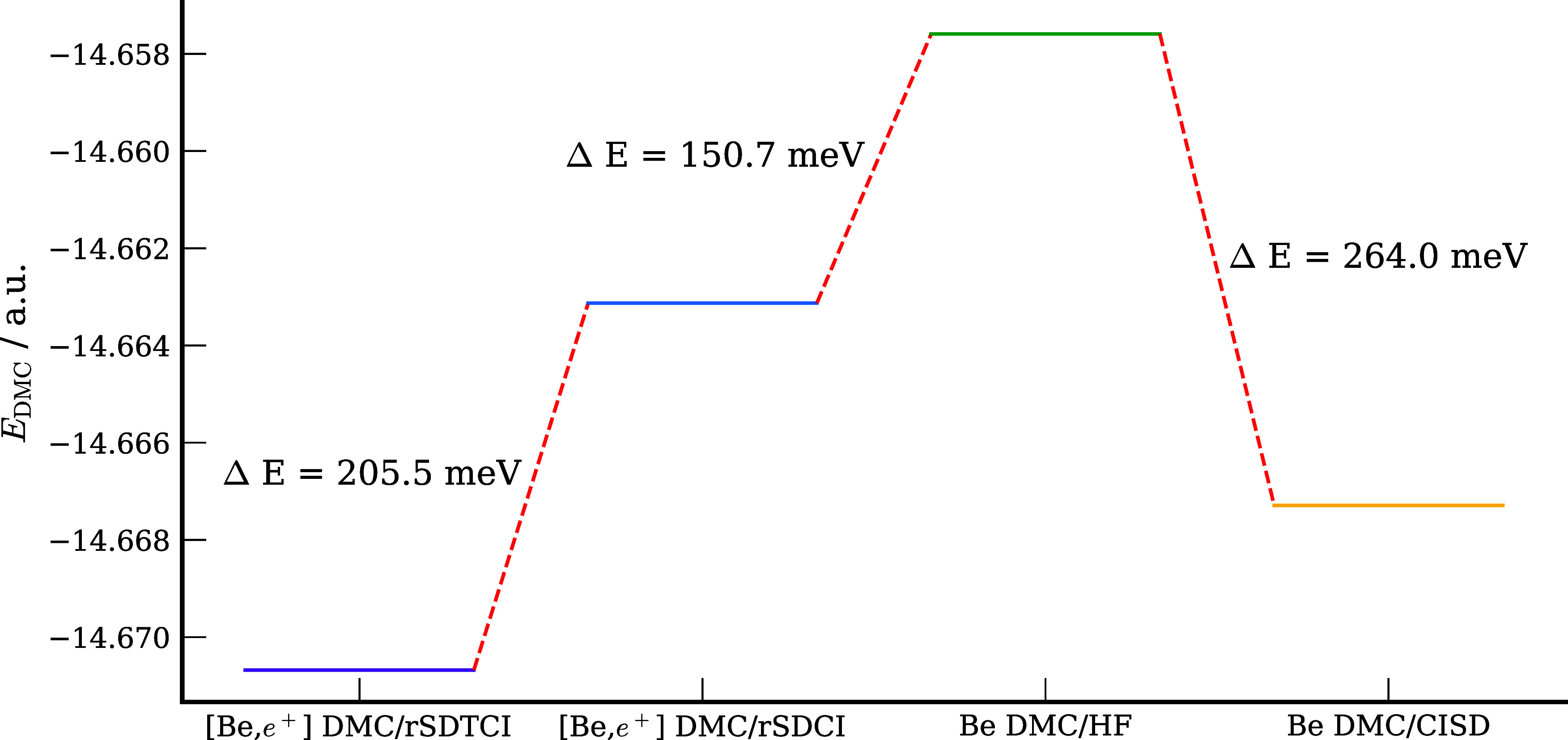
DMC energies of Be and [Be,e^+^] with various trial wave functions.

Figure 5 reports the DMC energies of Be with the HF and FCI trial wave functions as well as the DMC energies of [Be;e^+^] using rSDCI and FCI trial wave functions. It is seen from this figure that adoption of the FCI trial function for Be lowers its DMC energy by 264 meV compared to the result obtained using the HF trial wave function. The DMC/rSDCI energy of [Be;e^+^] is 151 meV below that of Be from the DMC/HF calculations. The energy of [Be;e^+^] is further lowered by 206 meV upon adoption of the FCI trial wave function. Thus, in the presence of the positron the inclusion of double excitations from the electronic 2s orbital makes a smaller impact on the nodal surface than for the isolated Be atom.

**Figure 6:**
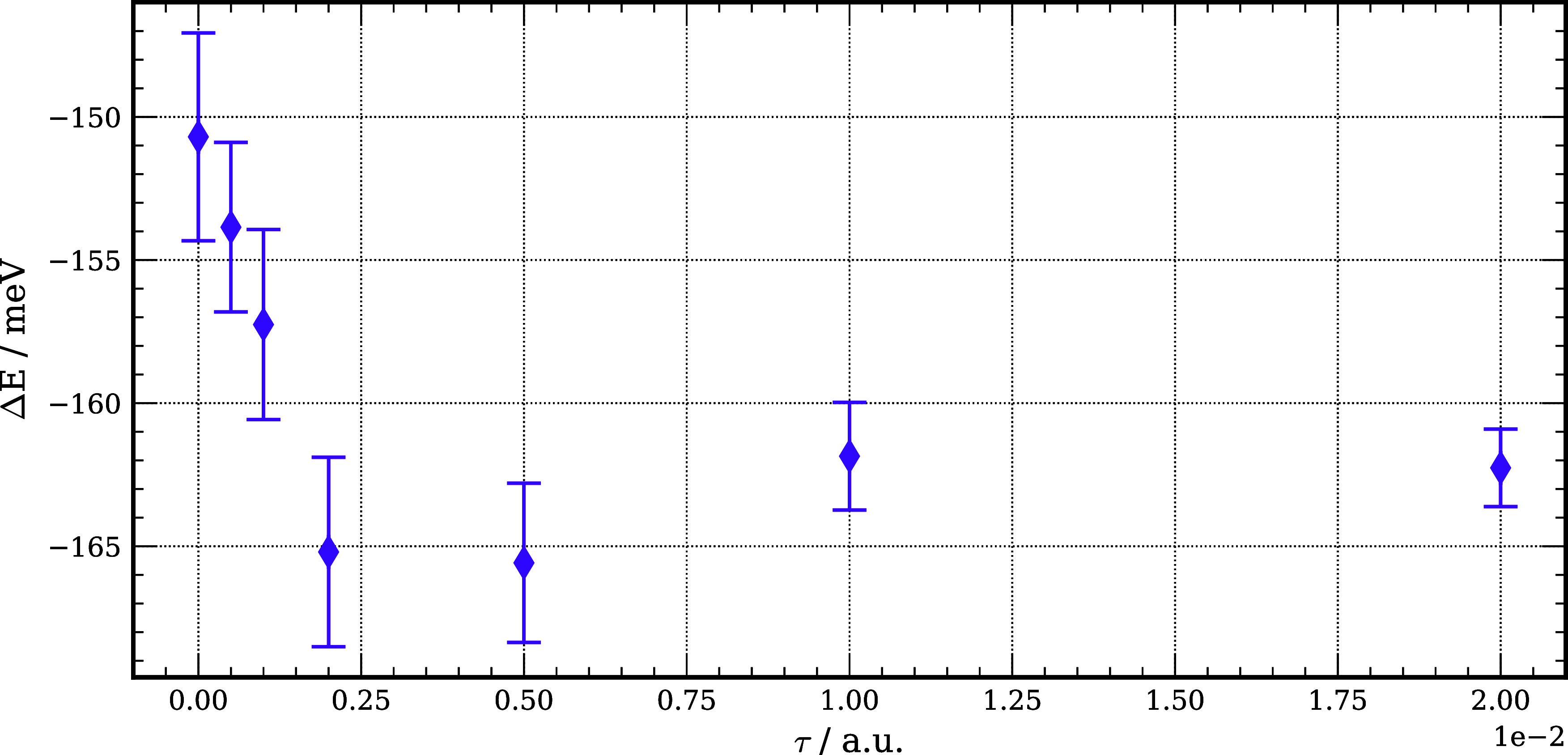
PA of Be calculated using DMC with a rSDCI multideterminant trial wave function for the positron complex and a HF trial function for the atom as a function of the time step size.

We additionally comment on the issue of choice of time steps for energy differences, a topic with wide implications for the quantum Monte Carlo (QMC) field. It has been found that for excitonic binding energies, one can use rather large time steps and still accurately resolve the energy differences in DMC calculations.^162^ With this in mind, we also carried out DMC calculations on Be and [Be;e^+^] using with larger time steps than employed for the results reported above, with the energies at the various time steps being reported in Figure 6. The large time step calculations are found to give a PA 11–14 meV larger than that obtained by extrapolation of the energy differences at the smaller time steps, i.e., 0.002, 0.001, and 0.005 au. This leads us to recommend caution with regard to the size of the time steps employed in DMC calculations when resolving energy differences for systems with large relevant length scales as one encounters in positron complexes.

### 3.3 Be_2_ and Be_4_

We now turn to the binding of a positron to Be_2_ and Be_4_. For Be_2_ we used the experimental bond length of 2.453603 Å,^163^ while for Be_4_ we considered the tetrahedral structure with Be–Be bond lengths of 2.085 Å.^164^ The aug-cc-pVQZ^159^ basis set was employed for the electrons and a 8s5p3d3f1g basis set for the positron for [Be_2_;e+], while the cc-pVQZ basis set was employed for the electrons and 8*s*4p2d2f1g basis set was employed for the positron for [Be_4_;e^+^]. The positron basis sets for the calculations were formed by truncating the basis set developed for Be and are given in the SI. Much larger basis sets than these would be needed to converge the PAs from CI calculations to the complete basis set limit. However, as seen above for the Be atom, even basis sets that result in a CI value of the PA much smaller than the complete basis set limit can give a well converged value of the PA when used to expand the trial functions in DMC calculations.

The results of the HF and CI calculations for the PA of the beryllium dimer and tetramer are presented in Table 1. For the positron complex of Be_2_, both the rSDCI procedure and a rSDTCI procedure were employed. The later employs for the positron complex a rSDTCI wave function, with the triple excitations constrained to involve excitation from the positron orbital together with double excitations from the valence electronic orbitals, and a CISD calculation for Be_2_ in the absence of the positron. Note that these are the same excitation levels as employed above in the FCI calculation of the PA of the Be atom. For the positron complex of Be_4_, rSDTCI calculations would have been computationally prohibitive with the basis sets employed, and, as a result, only the rSDCI procedure was considered in that case.

As expected, in the HF approximation the positron does not bind to Be_2_ or Be_4_. In contrast, with the rSDCI approach the positron is calculated to be bound to Be_2_ and Be_4_ by 482 and 771 meV, respectively. For comparison, we note that the rSDCI value of the PA of the beryllium atom was 151 meV. Based on the results for Be, we expect the rSDCI approach to overestimate the PA values of Be_2_ and Be_4_ for the basis sets employed. Indeed, the rSDTCI calculations give a PA of Be_2_ of 94 meV, compared to the 482 meV rSDCI result. Due to the limitations of the basis sets used for these calculations, the PA of Be_2_ obtained from the rSDTCI calculation is expected to be considerably underestimated.

The results of the DMC calculations of the PAs of Be_2_ and Be_4_ are reported in Table 3. For both Be_2_ and Be_4_, the SD-DMC calculations using HF orbitals and those using for the positron complex NOs from the rSDCI calculation give similar values of the PAs with the latter calculations giving PAs of 482 and 665 meV for Be_2_ and Be_4_, respectively. The DMC calculations using the rSDCI wave function as the trial function for the positron complexes and the HF wave functions as the trial functions in the absence of the positron give PAs of 601 and 770 meV for Be_2_ and Be_4_, respectively. Based on the results for the Be atom, the DMC calculations using the rSDCI trial functions for the complex are expected to overestimate the PAs of Be_2_ and Be_4_. Indeed, the DMC calculations using the using the rSDTCI trial wave function for Be_2_ give a PA of 445 meV, close to the DMC result obtained using HF trial functions. Thus, for both Be and Be_2_, DMC calculations using SD trial functions for both neutral systems and their positron complexes and using the rSDTCI trial wave functions (rSDTCI for the positron complex and CISD for the positron-free systems) give similar values for the PAs. This suggests that for these trial wave functions the positron does not make a significant impact on the electron–electron nodal surface for these species. On the other hand, the positron is far too localized in the rSDCI procedure, and this apparently causes a change in the electron–electron nodal surface leading to over binding of the positron to Be and Be_2_ (and, presumably also, to Be_4_). While we were not able to carry out rSDTCI calculations on Be_4_, and hence do not have DMC results with such trial functions for this system, based on the results for Be and Be_2_, we expect the PA of Be_4_ to be close to 626 meV obtained from the DMC calculations using the HF trial functions.

**Table 3:** PAs (meV) of Be_2_ and Be_4_ from Present DMC Calculations

method^a^	Be_2_	Be_4_
DMC: SD/HF//SD/HF	456 ± 11	626 ± 14
DMC: SD/HF//SD/NO rSDCI	482 ± 10	665 ± 11
DMC: SD/CISD NO//SD/rSDTCI NO	493 ± 10	
DMC: SD/HF//MD/rSDCI	601 ± 8	777 ± 12
DMC: MD/CISD//MD/rSDTCI	445 ± 9	

a The wave functions used in the absence of and in the presence of the positron are indicated to the left and right of the double slash, respectively.

We conclude this section with a more detailed analysis of the PAs from the rSDCI calculations. One can describe the PA as being comprised of a sum of terms: the energy cost of localizing the positron, the electrostatic interaction of the localized positron with the electrons and nuclei of the atom or molecule, and the correlation effects between the localized positron and the electrons of the system. A similar energy decomposition analysis was employed in analyzing the stability of NVCB anions.^137^ This decomposition scheme employs a definition of correlation different from what is commonly employed, but we believe that this is appropriate when the HF wave function experiences collapse onto a DC level. In a model potential approach, it is possible to extract each of the contributions described above, but this is not feasible in an ab initio treatment. However, one can estimate the kinetic energy (KE) and electrostatic contributions together by calculating the energy of the single configuration wave function constructed from the NOs of the CI calculation. When using NOs from the rSDCI calculation this approach places [Be;e^+^] 652 meV energetically above Be. However, when using the NOs from the FCI calculation we see that the localization and electrostatic effects place the positron energetically only 174 meV above the isolated Be atom. Because the positron is too localized in the rSDCI calculations, the positron-electron correlation (as defined above) is considerably overestimated in the rSDCI calculations. However, as noted above, the DMC value of the PA of Be calculated using SD trial functions is essentially independent of which of these sets of orbitals is used. While this need not be the case for Be_2_ and Be_4_, our calculations show that for these species when using SD trial wave functions the extent of the occupied positron orbital has little effect on the electron–electron nodal surface. We further note that the lowering of the DMC energies of Be and [Be;e^+^] in going from the HF to FCI trial function are nearly identical, indicating that, at least for the Be atom, the presence of the positron does not significantly impact the nodal surface for exchange of electrons.

### 3.4 Mg Atom

For the calculations on Mg we employed the aug-cc-pVQZ^159^ basis set for the electrons and the same 11s8p6d6f3g positron basis set as employed for Be. For Mg, our calculations using the rSDCI and FCI methods give PAs of 386 and 167.8 meV, respectively. Both of these values are significantly smaller than Bromley and Mitroy’s extrapolated CI result of 463.7 meV.^157^ This discrepancy is not surprising given the fact that our basis sets for the electrons and positron employed angular momentum functions only through *l* = 4, while the basis set employed Bromley and Mitroy employed angular momentum functions through *l* = 10.^157^ Part of the discrepancy could also be due to the use of the frozen-core approximation in our calculation. Although the calculations of Bromley and Mitroy also correlated only the two valence electrons, they did include polarization potentials to describe the response of the core electrons to the valence electrons and the positron. However, the goal of our CI calculations was not to reproduce the results of the Bromley-Mitroy calculations but rather to generate trial wave functions for subsequent DMC calculations that are far less sensitive to the basis sets employed.

For Mg, the PA obtained from SD-DMC calculations is found to depend sensitively on the orbitals employed in the trial wave function for [Mg;e^+^], being 90, 471, and 344 meV when using HF, NOs from the rSDCI calculations, and NOs from the FCI calculations, respectively. Hence the degree of localization of the positron *s* orbital of [Mg;e^+^] makes a significant impact on the nodal surface for electron exchange. This is unlike the situation for Be, Be_2_, and Be_4_ where the choice of orbitals for the SD-DMC calculations proved to be relatively uninportant.

The DMC calculations using the rSDCI and FCI trial wave functions for Mg and [Mg;e^+^] give PA values of 449 and 360 meV, respectively. The latter value is about 25% smaller than the Bromley-Mitroy extrapolated FCI value. This indicates that there is still a sizable nodal surface error in our FCI trial wave function for [Mg;e^+^]. This may reflect the need to include in the trial wave function configurations that account for correlation effects involving the core orbitals of Mg.

**Figure 7:**
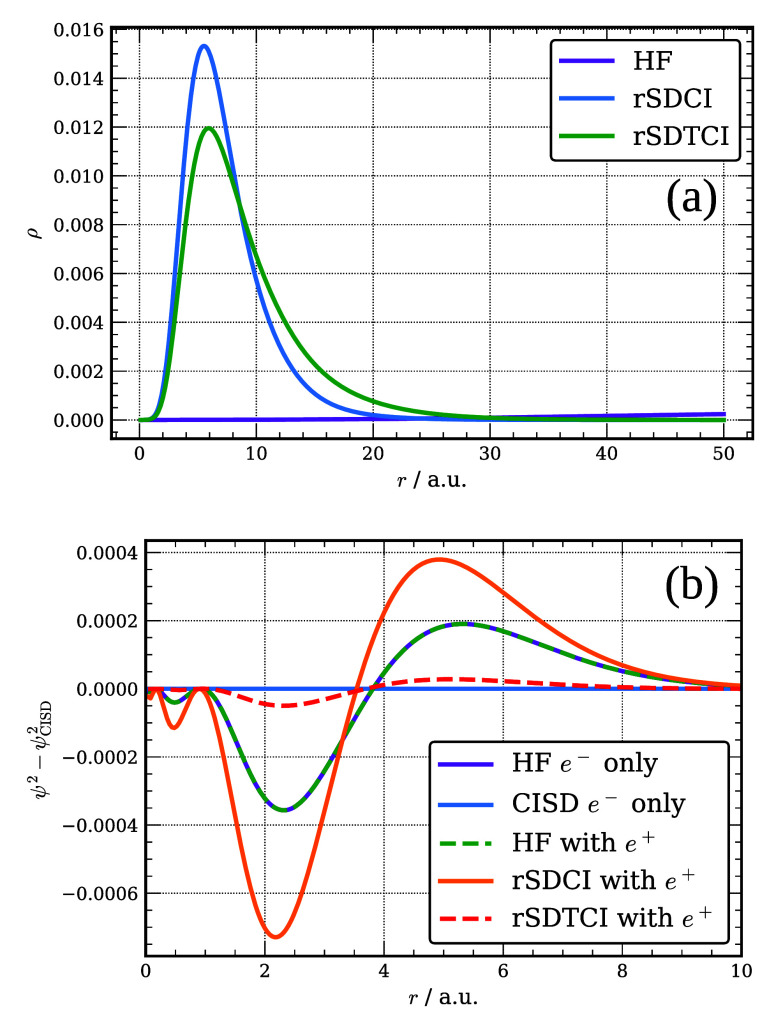
Radial distribution function of the Mg 1s positron orbital (a) and differences in the distribution of the 3s electron orbital of Mg and [Mg;e^+^] at various levels of theory relative to the CISD result for Mg (b).

In Figure 7, we report the radial distributions of the positron 1s natural orbital of the [Mg;e^+^] complex as described by various trial functions as well as how the charge distribution of the electronic 3s NO of [Mg;e^+^] and Mg varies with theoretical method. As seen from this figure, the positron 1s orbital from the HF calculations is extremely diffuse as it has collapsed onto a DC level. In contrast, the inclusion of correlation effects results in a localized 1s positron orbital, with its radial distribution peaking near 7.5 bohr in both the rSDCI and rSDTCI calculations, being somewhat less radially extended in the rSDCI calculations, consistent with the difference in the positron binding energies of the two CI calculations. It is also seen that inclusion of correlation effects leads to contraction of the 3s orbital of Mg. In the rSDCI calculations the presence of the positron causes the Mg 3s electronic orbital to become even more radially extended than that from the HF calculations on Mg. The main impact of the positron on the electron nodal surface is recovered using a single determinant trial wave function where the single determinant employs the NOs from the rSDCI calculations.

**Figure 8:**
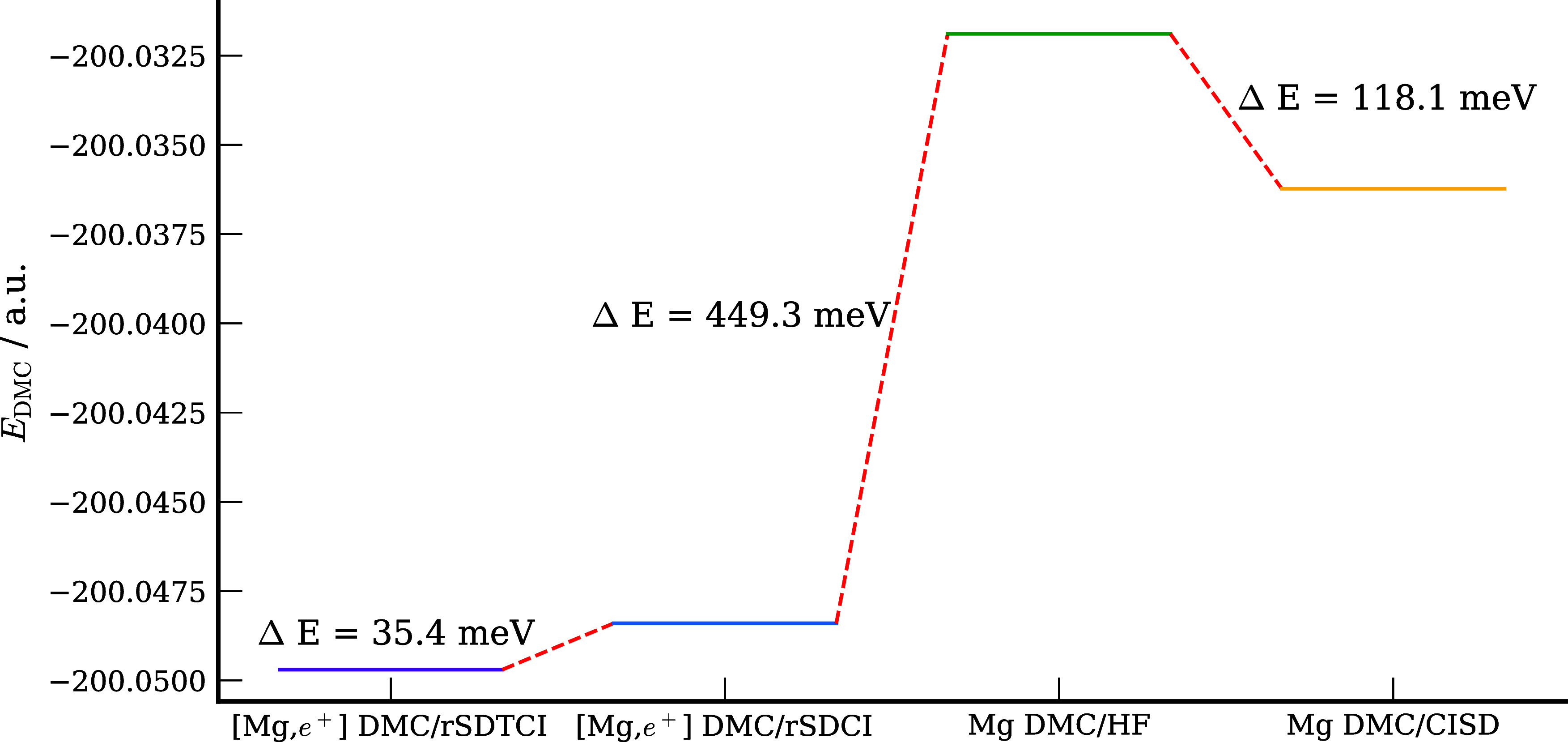
DMC energies of Mg and [Mg;e^+^] with various trial wave functions.

Figure 8 reports the DMC energies of Mg and [Mg;e^+^] obtained from the various DMC calculations. Comparison of this figure with the corresponding figure for Be (Figure 5) reveals that the lowering of the DMC energy of Mg upon adoption of the FCI trial function in place of the HF trial function is much smaller for Mg than for Be (118.1 meV for Mg vs 264.0 meV for Be). In other words, the nodal surface error for using a HF trial wave function is much greater for Be than for Mg. However, the energy lowering of the positron complex in going from the HF to the rSDCI trial wave functions is much larger for Mg than for Be (449.3 meV for Mg vs 150.7 meV for Be), while the energy lowering of the positron complex in going from the rSDCI trial wave function to the rSDTCI trial wave function is much smaller for Mg than for Be (35.4 meV for Mg vs 205.5 meV for Be). The greater lowering of the DMC energy of [Mg;e^+^] compared to [Be;e^+^] upon adoption of the rSDCI trial wave function is likely due to the much greater polarizability of Mg compared to Be.

### 3.5 Benzene

In our next example, we consider positron binding to the benzene molecule. A recent experimental study determined a value of 130 meV for the PA of benzene.^165^ A major challenge in characterizing positron binding to a polyatomic molecule is development of a suitable basis set for the positron. CI calculations employing electron and positron basis sets of the same size as that employed for Be for each of the atoms of benzene would be computationally prohibitive. However, as observed above, DMC calculations are far less sensitive to the basis set employed than are CI methods. With that in mind, in carrying out rSDCI calculations on the [benzene;e^+^] complex, we used the aug-cc-pVDZ basis set for the electrons, and an 8s4p2d2f2g GTO basis set at the center of the ring as well as 4s4p1d and 1s sets on the C and H atoms, respectively. The values of the PA of benzene obtained from our calculations as well as those from recent calculations using diagrammatic Green’s function (GF) based approach^166^ and from a neural network variational Monte Carlo (NNVMC) approach^167^ are summarized in Table 4.

**Table 4:** PA of Benzene Calculated Using Various Methods

method^a^	PA (meV)
HF//HF^b^	–41
HF//rSDCI^b^	77
GF^c^	116
Ferminet NNVMC^d^	–69
Ferminet NNVMC variance matched^d^	110 ± 10
DMC(SD/HF//SD/HF)^b^	–3 ± 26
DMC(SD/HF//SD/rSDCI NO)^b^	–24 ± 26
DMC(SD/HF//MD/rSDCI)^b^	137 ± 24

a The wave functions used in the absence of and in the presence of the positron are indicated to the left and right of the double slash, respectively.

b Present work.

c Hofierka et al.^166^

d Cassella et al.^167^

As expected, the HF calculations do not bind the positron to benzene. The rSDCI calculations bind the positron by 77 meV, about 1.7 times smaller than the experimental value of the PA of benzene. This is in contrast to contrast to our results for Be and Be_2_, for which the rSDCI approach gives PAs larger than or comparable to the best theoretical estimates. We attribute this to deficiencies in the electron and positron basis sets that we have used for benzene. However, the DMC calculations employing single determinant trial function, whether using for the positron complex the HF orbitals or the NOs from the rSDCI calculation, fail to bind the positron to benzene. Significantly, DMC calculations using as trial wave functions the rSDCI multideterminant expansion for [benzene;e+] and a SD of HF orbitals for the isolated benzene yield a PA of 137 ± 24 meV, which is in excellent agreement the experimental results.

Our DMC value of the PA of benzene is larger than previous ab initio results of 116 and 110 ± 10 meV using GF^166^ and a NNVMC^167^ calculations, respectively, but when allowing for the statistical errors, is consistent with these results. The values of the PAs from the Green’s function and VMC calculations are expected to be much more sensitive to the basis set than are the DMC results. Thus, it is likely that values of the PAs obtained using the Green’s function and VMC calculations are slightly underestimated due to the limitations of the basis sets employed.

### 3.6 Carbon Disulfide (CS_2_)

The final system studied was CS_2_ for which the value of the PA determined experimentally is 75 ± 10 meV.^168^ For this system we used the experimental geometry of CS_2_ with a CS bond length of 1.5562 Å.^169^ The aug-cc-pVTZ basis set was employed for the electrons and 16s9p6d4f and 7s5p3d2f basis sets, centered on the C and S atoms, respectively, were used for the positron. The positron basis set is given in the SI. The rSDCI calculations with this basis set is found to bind the positron by 34 meV. DMC calculations using the rSDCI trial wave functions give a PA of 87 ± 17 meV, which, allowing for the uncertainty in the experimental result and the statistical error in the DMC value, represents reasonable agreement between the DMC and experimental values. It would be desirable to extend these calculations in a future study to perform a extrapolation to zero time step.

## 4 Conclusions

We describe in this paper a flexible SCF and CI program, Polyquant, for describing systems with two or more distinct quantum particles. We use this code to develop trial wave functions for DMC calculations of positron binding energies for Be, Be_2_, Be_4_, Mg, CS_2_, and benzene.

Dynamic correlation associated with the e^+^/e^–^ cusp plays an exceptionally large role in the interaction of positrons with atoms and molecules. When using standard wave function based methods with Gaussian basis sets, very high (up to *l* ≅ 15) angular momentum functions are required to converge the energies of positron complexes, making such calculations of the positron affinities prohibitive for polyatomic systems. An attractive alternative is the use of the real-space diffusion Monte Carlo method, in which basis sets are introduced to represent a trial wave function to enforce the nodal surface for exchange of electrons. As long as the nodal surface is well described, DMC energies are relatively insensitive to the choice of basis set.

In this work we focused on the binding of a positron to uncharged, nonpolar systems where the binding is dominated by correlation effects. This introduces a new challenge to the calculations, namely that the simplest trial functions–a product of a positron orbital and a Slater determinant for the electrons–does not bind the positron. We demonstrate that in some cases–e.g., Be, Be_2_, Be_4_, and Mg – DMC calculations can overcome this limitation in the trial wave function. However, the situation is found to be different for benzene, for which DMC calculations using HF trial wave functions do not bind the positron. We attribute this to the limitations of the basis set employed. In addition, it is demonstrated that the rSDCI procedure provides effective, reasonably compact trial functions for DMC calculations on positron complexes, although for Be and Mg this approach is found to overbind the positron. Our DMC calculations on benzene using the rSDCI trial function for the positron complex and the HF wave function for the isolated benzene molecule give a PA in close agreement with the experimental value.

## Supporting Information Available

Basis sets used for the positron containing compounds (PDF)
