# Supplementary material for: Capturing Correlation Effects in Positron Binding to Atoms and Molecules
Source: J Chem Theory Comput. 2024 Sep 17;20(22):9879–93. doi: 10.1021/acs.jctc.4c00727 (PMC11603616; doi:10.1021/acs.jctc.4c00727)
Supplement: Supplementary file 1 — ct4c00727_si_001.pdf [file ct4c00727_si_001.pdf]

Supporting Information for Capturing Correlation Effects in Positron Binding to Atoms and Molecules

Shiv Upadhyay,[†,‡] Anouar Benali,[¶] and Kenneth D. Jordan[*,‡]

†*Department of Chemistry, University of Washington, Seattle, WA 98195*

‡*Department of Chemistry, University of Pittsburgh, Pittsburgh, PA 15218*

¶*Computational Sciences Division, Argonne National Laboratory, Argonne, IL 60439*

E-mail: jordan@pitt.edu

Contents

S1 Positron Basis sets **S1**

S1.1 Be basis set S2

S1.2 Be_2 basis set S3

S1.3 Be_4 basis set S4

S1.4 Mg basis set S4

S1.5 Benzene basis set S6

S1.6 HCN basis set S7

S1.7 CS2 basis set S8

S1 Positron Basis sets

All positron basis sets are provided in the Gaussian basis set format. The basis sets used for the electronic space are specified in the main text.

S1.1 Be basis set

```
****
Be     0
s   1   1.0
      3.2476950000     1
s   1   1.0
      1.0825650000     1
s   1   1.0
      0.3608550000     1
s   1   1.0
      0.1202850000     1
s   1   1.0
      0.0400950000     1
s   1   1.0
      0.0133650000     1
s   1   1.0
      0.0044550000     1
s   1   1.0
      0.0014850000     1
s   1   1.0
      0.0004950000     1
s   1   1.0
      0.0001650000     1
s   1   1.0
      0.0000550000     1
p   1   1.0
      1.0980089379     1
p   1   1.0
      0.3660029793     1
p   1   1.0
      0.1220009931     1
p   1   1.0
      0.0406669977     1
p   1   1.0
      0.0135556659     1
p   1   1.0
      0.0045185553     1
p   1   1.0
      0.0015061851     1
p   1   1.0
      0.0005020617     1
d   1   1.0
      0.3660030036     1
d   1   1.0
      0.1220010012     1
d   1   1.0
      0.0406670004     1
d   1   1.0
      0.0135556668     1
d   1   1.0
      0.0045185556     1
d   1   1.0
```

```
            0.0015061852        1
f    1        1.0
            0.3660030036        1
f    1        1.0
            0.1220010012        1
f    1        1.0
            0.0406670004        1
f    1        1.0
            0.0135556668        1
f    1        1.0
            0.0045185556        1
f    1        1.0
            0.0015061852        1
g    1        1.0
            0.3660030036        1
g    1        1.0
            0.1220010012        1
g    1        1.0
            0.0406670004        1
****
```

S1.2 Be_2 basis set

```
****
Be          0
s    1        1.0
            3.2476950000        1
s    1        1.0
            1.0825650000        1
s    1        1.0
            0.3608550000        1
s    1        1.0
            0.1202850000        1
s    1        1.0
            0.0400950000        1
s    1        1.0
            0.0133650000        1
s    1        1.0
            0.0044550000        1
s    1        1.0
            0.0014850000        1
p    1        1.0
            1.0980089379        1
p    1        1.0
            0.3660029793        1
p    1        1.0
            0.1220009931        1
p    1        1.0
            0.0406669977        1
p    1        1.0
            0.0135556659        1
d    1        1.0
            0.3660030036        1
```

```
d    1      1.0
            0.1220010012        1
d    1      1.0
            0.0406670004        1
f    1      1.0
            0.3660030036        1
f    1      1.0
            0.1220010012        1
f    1      1.0
            0.0406670004        1
g    1      1.0
            0.3660030036        1
****
```

S1.3 Be_4 basis set

```
****
Be         0
s    1      1.0
            3.2476950000        1
s    1      1.0
            1.0825650000        1
s    1      1.0
            0.3608550000        1
s    1      1.0
            0.1202850000        1
s    1      1.0
            0.0400950000        1
s    1      1.0
            0.0133650000        1
s    1      1.0
            0.0044550000        1
s    1      1.0
            0.0014850000        1
p    1      1.0
            1.0980089379        1
p    1      1.0
            0.3660029793        1
p    1      1.0
            0.1220009931        1
p    1      1.0
            0.0406669977        1
d    1      1.0
            0.3660030036        1
f    1      1.0
            0.3660030036        1
****
```

S1.4 Mg basis set

```
****
Mg         0
```

```
s   1    1.0
       3.2476950000        1
s   1    1.0
       1.0825650000        1
s   1    1.0
       0.3608550000        1
s   1    1.0
       0.1202850000        1
s   1    1.0
       0.0400950000        1
s   1    1.0
       0.0133650000        1
s   1    1.0
       0.0044550000        1
s   1    1.0
       0.0014850000        1
s   1    1.0
       0.0004950000        1
s   1    1.0
       0.0001650000        1
s   1    1.0
       0.0000550000        1
p   1    1.0
       1.0980089379        1
p   1    1.0
       0.3660029793        1
p   1    1.0
       0.1220009931        1
p   1    1.0
       0.0406669977        1
p   1    1.0
       0.0135556659        1
p   1    1.0
       0.0045185553        1
p   1    1.0
       0.0015061851        1
p   1    1.0
       0.0005020617        1
d   1    1.0
       0.3660030036        1
d   1    1.0
       0.1220010012        1
d   1    1.0
       0.0406670004        1
d   1    1.0
       0.0135556668        1
d   1    1.0
       0.0045185556        1
d   1    1.0
       0.0015061852        1
f   1    1.0
       0.3660030036        1
f   1    1.0
       0.1220010012        1
f   1    1.0
```

```
        0.0406670004        1
f   1     1.0
        0.0135556668        1
f   1     1.0
        0.0045185556        1
f   1     1.0
        0.0015061852        1
g   1     1.0
        0.3660030036        1
g   1     1.0
        0.1220010012        1
g   1     1.0
        0.0406670004        1
****
```

S1.5 Benzene basis set

```
****
X       0
s   1     1.0
        1.0825650000        1
s   1     1.0
        0.3608550000        1
s   1     1.0
        0.1202850000        1
s   1     1.0
        0.0400950000        1
s   1     1.0
        0.0133650000        1
s   1     1.0
        0.0044550000        1
s   1     1.0
        0.0014850000        1
s   1     1.0
        0.0004950000        1
p   1     1.0
        0.3660029793        1
p   1     1.0
        0.1220009931        1
p   1     1.0
        0.0406669977        1
p   1     1.0
        0.0135556659        1
d   1     1.0
        0.1220009931        1
d   1     1.0
        0.0406669977        1
f   1     1.0
        0.1220009931        1
f   1     1.0
        0.0406669977        1
g   1     1.0
        0.1220009931        1
```

```
g    1      1.0
        0.0406669977       1
****
C       0
S      1      1.00
        3.600000D–01                  1.000000D+00
S      1      1.00
        1.200000D–01                  1.000000D+00
S      1      1.00
        4.000000D–02                  1.000000D+00
S      1      1.00
        1.333333D–03                  1.000000D+00
S      1      1.00
        4.444444D–04                  1.000000D+00
P      1      1.00
        3.600000D–01                  1.000000D+00
P      1      1.00
        1.200000D–01                  1.000000D+00
P      1      1.00
        4.000000D–02                  1.000000D+00
P      1      1.00
        1.333333D–03                  1.000000D+00
D      1      1.00
        1.200000D–01                  1.000000D+00
****
H       0
S      1      1.00
        1.000000D+00                  1.000000D+00
****
```

S1.6 HCN basis set

```
****
C       0
S      1      1.00
        3.600000D–01                  1.000000D+00
S      1      1.00
        1.200000D–01                  1.000000D+00
S      1      1.00
        4.000000D–02                  1.000000D+00
S      1      1.00
        1.333333D–03                  1.000000D+00
S      1      1.00
        4.444444D–04                  1.000000D+00
P      1      1.00
        3.600000D–01                  1.000000D+00
P      1      1.00
        1.200000D–01                  1.000000D+00
P      1      1.00
        4.000000D–02                  1.000000D+00
```

```
P     1    1.00
        1.333333D–03              1.000000D+00
D     1    1.00
        1.200000D–01              1.000000D+00
****
N      0
S     1    1.00
        3.600000D–01              1.000000D+00
S     1    1.00
        1.200000D–01              1.000000D+00
S     1    1.00
        4.000000D–02              1.000000D+00
S     1    1.00
        1.333333D–03              1.000000D+00
S     1    1.00
        4.444444D–04              1.000000D+00
P     1    1.00
        3.600000D–01              1.000000D+00
P     1    1.00
        1.200000D–01              1.000000D+00
P     1    1.00
        4.000000D–02              1.000000D+00
P     1    1.00
        1.333333D–03              1.000000D+00
D     1    1.00
        1.200000D–01              1.000000D+00
****
H      0
S     1    1.00
        1.000000D+00              1.000000D+00
****
```

S1.7 CS2 basis set

```
****
C      0
S     10    1.00
        8.236000D+03              5.310000D–04
        1.235000D+03              4.108000D–03
        2.808000D+02              2.108700D–02
        7.927000D+01              8.185300D–02
        2.559000D+01              2.348170D–01
        8.997000D+00              4.344010D–01
        3.319000D+00              3.461290D–01
        9.059000D–01              3.937800D–02
        3.643000D–01             –8.983000D–03
        1.285000D–01              2.385000D–03
S     1    1.00
        9.059000D–01              1.000000D+00
S     10    1.00
```

```
          8.236000D+03          -1.130000D-04
          1.235000D+03          -8.780000D-04
          2.808000D+02          -4.540000D-03
          7.927000D+01          -1.813300D-02
          2.559000D+01          -5.576000D-02
          8.997000D+00          -1.268950D-01
          3.319000D+00          -1.703520D-01
          9.059000D-01           1.403820D-01
          3.643000D-01           5.986840D-01
          1.285000D-01           3.953890D-01
S    1    1.00
          1.285000D-01           1.000000D+00
S    1    1.00
          0.0440200              1.0000000
P    1    1.00
          3.827000D-01           1.000000D+00
P    5    1.00
          1.871000D+01           1.403100D-02
          4.133000D+00           8.686600D-02
          1.200000D+00           2.902160D-01
          3.827000D-01           5.010080D-01
          1.209000D-01           3.434060D-01
P    1    1.00
          1.209000D-01           1.000000D+00
P    1    1.00
          0.0356900              1.0000000
D    1    1.00
          1.097000D+00           1.000000D+00
D    1    1.00
          3.180000D-01           1.000000D+00
D    1    1.00
          0.1000000              1.0000000
F    1    1.00
          7.610000D-01           1.0000000
F    1    1.00
          0.2680000              1.0000000
S   1   1.0
          0.00551239832656    1
S   1   1.0
          0.00220495933062    1
S   1   1.0
          0.00088198373225    1
S   1   1.0
          0.00035279349290    1
S   1   1.0
          0.00014111739712    1
S   1   1.0
          0.00005644695885    1
S   1   1.0
          0.00002257878354    1
S   1   1.0
          0.00000903151342    1
```

```
S   1     1.0
          0.00000361260537       1
S   1     1.0
          0.00000144504215       1
S   1     1.0
          0.00000057801686       1
P   1     1.0
          0.01378099581641       1
P   1     1.0
          0.00551239832656       1
P   1     1.0
          0.00220495933062       1
P   1     1.0
          0.00088198373225       1
P   1     1.0
          0.00035279349290       1
D   1     1.0
          0.05512398326563       1
D   1     1.0
          0.02204959330625       1
D   1     1.0
          0.00881983732250       1
F   1     1.0
          0.05512398326563       1
F   1     1.0
          0.02204959330625       1
****
S       0
S      15     1.00
          3.741000D+05               5.421400D-05
          5.605000D+04               4.208550D-04
          1.276000D+04               2.206980D-03
          3.615000D+03               9.192580D-03
          1.183000D+03               3.211230D-02
          4.288000D+02               9.466830D-02
          1.678000D+02               2.236300D-01
          6.947000D+01               3.743930D-01
          2.984000D+01               3.291080D-01
          1.272000D+01               8.470380D-02
          5.244000D+00               4.408510D-04
          2.219000D+00               1.648270D-03
          7.767000D-01              -6.223320D-04
          3.490000D-01               3.013060D-04
          1.322000D-01              -8.412900D-05
S      15     1.00
          3.741000D+05              -1.498370D-05
          5.605000D+04              -1.161980D-04
          1.276000D+04              -6.115830D-04
          3.615000D+03              -2.553700D-03
          1.183000D+03              -9.087080D-03
          4.288000D+02              -2.770450D-02
          1.678000D+02              -7.200200D-02
```

	6.947000D+01	−1.464390D−01
	2.984000D+01	−1.951500D−01
	1.272000D+01	8.191930D−03
	5.244000D+00	5.166010D−01
	2.219000D+00	5.421780D−01
	7.767000D−01	6.884300D−02
	3.490000D−01	−9.180720D−03
	1.322000D−01	2.268320D−03
S	1 1.00	
	7.767000D−01	1.000000D+00
S	15 1.00	
	3.741000D+05	4.350660D−06
	5.605000D+04	3.371400D−05
	1.276000D+04	1.776740D−04
	3.615000D+03	7.411160D−04
	1.183000D+03	2.645910D−03
	4.288000D+02	8.074870D−03
	1.678000D+02	2.122760D−02
	6.947000D+01	4.383230D−02
	2.984000D+01	6.127160D−02
	1.272000D+01	−3.615100D−03
	5.244000D+00	−2.045100D−01
	2.219000D+00	−3.818710D−01
	7.767000D−01	8.268440D−02
	3.490000D−01	7.141470D−01
	1.322000D−01	3.937910D−01
S	1 1.00	
	1.322000D−01	1.000000D+00
S	1 1.00	
	0.0497000	1.0000000
P	9 1.00	
	5.744000D+02	2.422640D−03
	1.358000D+02	1.927960D−02
	4.319000D+01	8.854010D−02
	1.587000D+01	2.546540D−01
	6.208000D+00	4.339840D−01
	2.483000D+00	3.549530D−01
	8.688000D−01	6.189410D−02
	3.229000D−01	−5.029770D−03
	1.098000D−01	2.098130D−03
P	1 1.00	
	8.688000D−01	1.000000D+00
P	9 1.00	
	5.744000D+02	−6.201020D−04
	1.358000D+02	−4.938820D−03
	4.319000D+01	−2.326470D−02
	1.587000D+01	−6.851950D−02
	6.208000D+00	−1.238960D−01
	2.483000D+00	−9.694990D−02
	8.688000D−01	2.282150D−01
	3.229000D−01	5.693940D−01

```
         1.098000D–01                3.663020D–01
P    1     1.00
         1.098000D–01                1.000000D+00
P    1     1.00
         0.0351000                   1.0000000
D    1     1.00
         8.190000D–01                1.000000D+00
D    1     1.00
         2.690000D–01                1.000000D+00
D    1     1.00
         0.1010000                   1.0000000
F    1     1.00
         5.570000D–01                1.0000000
F    1     1.00
         0.2180000                   1.0000000
****
```
